# Perspectives of older patients on the qualities which define a “good family nurse”: A qualitative study

**DOI:** 10.1002/nop2.456

**Published:** 2020-02-14

**Authors:** Ludmila Marcinowicz, Ewa Taranta

**Affiliations:** ^1^ Department of Primary Health Care Medical University of Bialystok Bialystok Poland

**Keywords:** family nurse, good nurse, nursing, older people, primary health care

## Abstract

**Aim:**

To explore what the term “good family nurse” means to older patients.

**Design:**

A descriptive qualitative study design was used, and a purposive sampling method was adopted.

**Methods:**

Semi‐structured interviews were conducted with 21 patients aged 65 years and older who were receiving primary care in Bialystok (Poland). The interviews were recorded and then transcribed in verbatim. The data were analysed using content analysis. Data were collected between February 2017 and December 2018.

**Results:**

We identified six main categories of qualities that define a “good family nurse”. These are as follows: (a) personal traits and attributes (sex and individual characteristics and behaviours not directly related to nursing); (b) providing care (caring attitude and patient support); (c) communicating with the patient (the ability to listen and inform the patient); (d) professional competence (knowledge, professional experience and good technical skills); (e) ethical attitude (respect, patience and vocation); and (f) availability (the frequency and duration of home visits, organization of the doctor's appointments).

## 1 INTRODUCTION

All over the world, the population is ageing, forcing healthcare systems to devise effective care strategies for older adults which meet their needs and expectations. In many countries, healthcare systems are increasingly turning to local involvement for care planning and providing community‐based services (WHO, 2015). Scientific research has been fundamental to the improvement of human health and the World Health Organization recommends that research be undertaken to improve the quality of health services and, consequently, the health and well‐being of society (WHO, 2013).

The role of the patient in evaluating health care and contributing to its quality is well documented in the medical literature (Browne, Roseman, & Shaller, 2010; Gleeson et al., 2016; Marcinowicz, Gugnowski, Strumiło, & Chlabicz, 2015). A literature review by Longtin et al. (2010) suggests that patient participation can both improve care in the management of long‐term illness and modify the behaviour of healthcare workers.

According to elderly patients, a good nurse should have the necessary technical competence and comprehensive knowledge, as well as psychosocial skills to provide care (Van der Elst, Dierckx de Casterle, & Gastmans, 2012). Using data from patients' experiences is an expression of patient‐focused care and can be an important strategy for transforming nursing practice. Therefore, it is important to carry out in‐depth qualitative research to understand better the phenomenon of a “good nurse”. The progressive ageing of the population is associated with an increase in the number of older people (≥65 years) as primary care patients. Understanding the patients' perspective on a “good family nurse” can improve the quality of health care for these patients.

## 2 BACKGROUND

In Poland, the family nurse is a member of the primary healthcare (PHC) team, which also includes a doctor and midwife. According to Ordinance of the Minister of Health (2016), a family nurse provides care for healthy, ill and disabled patients regardless of sex and age, except for infants and babies up to two months of age. The family nurse plans and provides nursing care for patients and their families, delivering a range of services, from health promotion and disease prevention to nursing, diagnostic, therapeutic and rehabilitation services. Health services and nursing activities carried out by a family nurse in the patient's place of residence include among others: participation in the implementation of preventive health programs, health education, performing physical examinations, education in the field of self‐care in relation to the sick person and their family (caregivers). The tasks of a family nurse also involve assistance in acquiring rehabilitation equipment, auxiliary equipment and orthopaedic supplies necessary for care and rehabilitation; recognizing domestic violence and other social pathologies and intervening in family crisis situations, and organization of institutional assistance (e.g. referring the patient to social welfare centres). Health services and nursing activities carried out by the family nurse at the surgery and vaccination point are among others: counselling for healthy persons, sick persons and those at risk of illness and administering preventive vaccinations. Furthermore, a family nurse's tasks include other medical activities related to work organization, for example preparing the surgery (i.e. doctor's office) and treatment equipment for patients and management of medicines, medical equipment and dressing materials.

In Poland, patients have the right to choose their family nurse, as they do their family doctor and family midwife; however, they can do so no more than twice a year. Each patient chooses the nurse by filling in a declaration form available from the PHC centre. One family nurse can provide care for up to 2,500 patients.

Understanding how older patients perceive the term “good family nurse” is of great social interest and can help improve the quality of nursing services in PHC. A previous analysis of this concept (Van der Elst et al., 2012) was a review of the literature from 1990–2010 and considered “the good nurse” in a general sense. Our work focuses on the characteristics of a good family nurse in the context of the PHC system. It is important because patients' expectations regarding healthcare providers may change over time.

We have assumed that the notion of doing good, being good and acting on the good is related to the normative practice and standards of the profession of nursing (Smith & Godfrey, 2002).

## 3 METHODS

### 3.1 Aim and design

The aim of this study was to explore what the term “good family nurse” means to older patients and what attributes comprise this concept.

### 3.2 Design

This study adopted a descriptive qualitative approach using face‐to‐face interviews (Sandelowski, 2000). The qualitative research was carried out using the interview technique as relevant to explore patient experiences and opinions.

### 3.3 Sampling

Patients aged 65 and above at six outpatient clinics, representing both sexes and different levels of experience in receiving care of a family nurse, were purposively recruited for the study. The purposive sampling strategy with maximum variation was employed to collect the opinions of patients of different ages and sexes and with different health problems. Maximum variation sampling is one of the most frequently used kinds of purposeful sampling in qualitative nursing research and allows researchers to explore a target phenomenon across demographically varied cases (Sandelowski, 1995).

### 3.4 Participants

All the participants were cared for by a family nurse of their choice. Of the 21 participants, 12 (57%) were female and 9 (43%) were male. The participants ranged in age from 65–88 years (mean = 72 years). They had different levels of education, different marital status, different health problems and experience in receiving care from a family nurse (Table 1). Participants perceived family nurse services in accordance with nurses' tasks: health education services, disease prevention services, diagnostic services, nursing services, rehabilitation and therapeutic services.

**Table 1:** Characteristics of the participants

	Sex	Age	Education	Marital status	Main health problem	Using the services of the family nurse
1.	M	72	Technical	Married	Osteoarthritis	Once a month
2.	F	68	Secondary	Married	Hypertension	Every 3 months
3.	F	78	Elementary	Married	Spine pain	Once a month
4.	F	70	University	Married	Spine pain	Every 3 months
5.	F	73	Secondary	Widow	Knee endoprosthesis	Every 2 months
6.	M	66	Secondary	Married	Diabetes	Once a year
7.	M	83	Secondary	Married	Pacemaker	Once a month
8.	F	65	Secondary	Married	Bronchial asthma	Once a year
9.	F	68	Technical	Single	Gout	Once a month
10.	F	66	Secondary	Married	Hypothyroidism	Every 3 months
11.	F	70	Secondary	Married	Osteoarthritis	Once a year
12.	F	68	Secondary	Single	Hypertension	Once a year
13.	M	78	Elementary	Married	Diabetes	Every 6 months
14.	M	65	Technical	Married	Depression	Once a year
15.	F	80	Secondary	Widow	Wound on the back	Twice a week
16.	F	88	Secondary	Married	Bedsores	Once a week
17.	M	83	Elementary	Married	Pacemaker	Every 6 months
18.	M	68	Technical	Married	Rheumatism	Once a year
19.	M	71	Secondary	Married	Prostate hypertrophy	Every 3 months
20.	K	65	Technical	Widow	Hyperthyroidism	Once a year
21.	M	73	Technical	Married	Heart disease	Once a year

### 3.5 Data collection

The interviews were performed at a time and place convenient for each participant. Sixteen interviews were conducted at the patients' homes and 5 interviews at outpatient clinics. All the interviews were conducted in Polish by the same interviewer (paper contributor ET), who is trained in qualitative research. The interview guide was developed based on literature (Catlett & Lovan, 2011; Smith & Godfrey, 2002). The participants were asked the following questions: Please tell us about your latest visit to the clinic. How often do you use the services of a family nurse? What does the term “good family nurse” mean for you? What should a “good family nurse” be like? The interviews lasted 20–40 min were audio‐recorded and later transcribed by the interviewer. Three patients (two men and one woman) refused to participate in the study without specifying a reason. Data were collected between February 2017 and December 2018.

### 3.6 Ethical considerations

Ethical approval was granted by the Bioethics Committee of the Medical University of Bialystok. Participation in the study was voluntary. Each patient was asked for consent at the beginning of the interview, and an oral informed consent was obtained from participants.

### 3.7 Data analysis

The Graneheim and Lundman (2004) approach to qualitative content analysis was used. After printing, all the transcripts were read through several times and then analysed and manually coded by two researchers (the paper's authors). The text about the participants' opinions on a “good family nurse” was extracted and compiled into a single text document, which constituted the unit of analysis. Next, the text was divided into meaning units and sorted into 15 subcategories and six categories, which constitute the manifest content. Any differences in coding or categorization were discussed by the two researchers until consensus was achieved. Theoretical saturation was achieved when the participants' responses repeated and when no new information emerged during coding (Strauss & Corbin, 1998).

### 3.8 Trustworthiness

Trustworthiness was ensured by means of purposive sampling. All participants were selected for the purpose of the study. The interviewer recorded field notes after each interview. The reliability of the analysis was ensured by the authors' reading and rereading the transcripts and by discussing and agreeing on the categories and subcategories. Divergent interpretations were discussed between the authors until a common set of categories was agreed.

Member checking was used to establish the validity of the researchers' interpretations of the data (Sandelowski, 1993). The report was shown to six study participants, who concurred with the results.

## 4 FINDINGS

### 4.1 Ways of understanding the term of “good family nurse”

We identified six categories of understanding of the term “good family nurse” as expressed by older patients: (a) personal traits and attributes; (b) providing care; (c) professional competence; (d) communicating with the patient; (e) ethical attitude; and (f) availability (Table 2).

**Table 2:** Categories, subcategories and units of meaning

Categories	Subcategories	Units of meaning
Personal traits and attributes	Sex Personal traits and attributes not directly related to the provision of care	Female Kind, polite Emotionally stable
Providing care	Caring attitude to the patient Support	Care for the patient Therapeutic relationship
Professional competence	Professional experience Good technical skills Professionalism	Work experience as a nurse Ability to give injections Knowledge of the job
Communicating with the patient	Informing the patient Listening Individual approach to the patient	Using language understandable to the patient Listening to what the patient has to say Using the patient's first name
Ethical attitude	Respect Patience Vocation	Treating people with respect Being open to human suffering Devotion to the job
Availability	Frequency and duration of home visits Organization of doctor's appointments	Regularity of home visits Devoting more time to patients Arranging doctor's appointments Registering patients

#### 4.1.1 Personal traits and attributes

For the participants, the term “good family nurse” was connected with the traditional perception of nursing performed by women, who are commonly attributed such qualities as warmth, calmness and self‐control. This category included the nurse's individual traits and behaviours that are not directly related to patient care but have an influence on the provision of care:

She should be a warm, sister‐like woman. I always call them sisters and I think she should be like a sister to me. (Participant 1)

The participants often pointed out that a nurse should be kind, polite and not be nervous.

They emphasized the need for such behaviour especially towards the elderly who cannot cope alone:

She's kind and polite and never gets angry if I come [to the clinic] so often. She lets me in and says of course I should come, because an older person cannot manage by himself. (Participant 15)

Some responses referred to an approach to the patient as having a therapeutic effect:

She is nice and kind, with a positive attitude to patients my age and older. A good word is better than drugs. (Participant 18)

Sometimes they emphasized that an elderly person needs special care and even love from a family nurse:

A good family nurse should be very nice for the patient. She shouldn't be rude or nervous. She shouldn't be so, you know, cold. She should approach a seriously ill person with gentleness, because such a person needs care and love. I think it should work this way. (Participant 10)

The participants were aware that the nurse's job is stressful, and they expressed the opinion that a good family nurse should be emotionally stable:

Calm, self‐controlled, not nervous. In this job you need self‐control. Of course, the patient is nervous, so the nurse should calm you down and make you feel comfortable. (Participant 3)

#### 4.1.2 Providing care

The most frequent statements in this category were “she cares for the patients,” “she provides support” etc., with emphasis on the fact that the patients are elderly, lonely people:

It means she cares for the patient properly. She assists patients, especially older ones. (Participant 3)

Some participants described a good family nurse as one who provides elderly people, especially lonely people, with care that takes into account their needs:

She provides good care. If a nurse comes to the home of a seriously ill patient, especially if the person lives alone and not only gives the injection but also talks to the person and asks what he or she needs, it gives them support. (Participant 19)

The participants' opinions on a good family nurse also took the form of expectations connected with the patient's health status. However, they also stressed the role of the family in providing care for the patient:

Considering my present health condition and my level of ability, I don't need any more care from the nurse than she is giving me now. What she does for me is enough. I don't know what it would look like if I were bed‐ridden at home. Then I would expect someone to bring me medicines if there was nobody available from my family. I don't know if it's possible, but I imagine it would be perfect this way. (Participant 4)

#### 4.1.3 Professional competencies

In this category, participants' responses concerning a good family nurse referred to her knowledge about diseases and drugs and patients expected advice in this regard. However, their responses suggested that nurses' knowledge actually came from doctors, not from formal nursing education:

I would like her to give me some advice if I need it, because she works with the doctor, so she knows a lot about diseases and drugs. She surely knows. (Participant 2)

The participants emphasized that the nurse should have good technical skills. This was reflected by their use of the term “professional” in their responses:

She's professional, she skillfully gives injections and takes blood samples. (Participant 12)

She should be good in giving injections painlessly, she should have a good hand, if you can say so. Because not every nurse is good in giving injections. Sometimes it's so painful when they do this and sometimes not. The nurse needs to be skillful. (Participant 1)

Professional experience is something every good family nurse should have. The participants were clear about this quality:

First of all, she must be professional. It should be a nurse with several years of work experience, knowing her job and doing it properly and diligently. (Participant 11)

#### 4.1.4 Communicating with the patient

The participants claimed that a good family nurse should have good communication skills, especially the skill of providing information in a simple and understandable way:

The ability to talk to the patient, so that the patient could understand what she [the nurse] is talking about. (Participant 6)

The participants also stressed the necessary skill of listening to the patient and not distancing herself from them. Older patients were sensitive to the nurse's tone of voice and the way the nurse addresses them. In addition, the participants are aware of the qualities they themselves may have because of their age and they expect the nurse to appreciate these things:

I wouldn't like her to shout at the patient if he doesn't know something, because I've met some nurses who do. Not in our clinic, but when I was in another one… Come on… I'd like her to listen to what I have to say and not to think I'm just jawing. I'm old so maybe I do jaw, but I shouldn't be afraid to approach her and ask her something. (Participant 4)

From the patient's perspective, it is important that the nurse knows her patients and addresses them personally, using their first names:

Only one nurse knows my name and uses it. Others treat me like a stranger. (Participant 5)

#### 4.1.5 Ethical attitude

Some participants defined a good family nurse by her respect for people, especially for the elderly and chronically ill. In addition, they considered smiling and cordiality to be important features of nursing care that strengthen the patient:

Respect for older and younger people alike. Respect for everyone. Everybody should be treated with respect and if a person is bed‐ridden and the nurse approaches him, giving smiles and showing warmth are also important. This is essential, because it gives the patient strength. (Participant 9)

The participants also mentioned other ethical behaviours, such as being open to human suffering.

A good family nurse is one with a soul. She's open to people's pain. And good to people. And kind. (Participant 17)

According to the participants, the nurse should like her job, because it is a necessary attribute for doing the job well. Participants stated that nurses need to have the vocation to do the job:

It seems to me a good family nurse must like and love her job, like in every other profession. You need to have a vocation. Otherwise, you cannot be good in it. If you don't like the job you do, how can you be good? This vocation is inborn. (Participant 21)

#### 4.1.6 Availability

In the “availability” category, the participants' responses referred to general availability of a family nurse as regards to home visits:

If you call a nurse to your home, she should come and care for the patient, for example give them an injection or provide professional advice. She should be available when you need her. (Participant 7)

Some responses suggested dissatisfaction with infrequent and short visits by a nurse, although the participants did not express it directly but rather indirectly and using plural forms. One participant expressed it on behalf of all patients:

We would all like nurse's visits to be much more frequent and we'd like her to devote even more time to us. But I don't know if this is possible in the situation of our health care. I suppose it's just wishful thinking. (Participant 19)

The responses of some participants had the form of expectations, stressing the situation of elderly people:

I would expect her to visit us more often, especially older people, because nurses don't come very often. (Participant 3)

Other responses referred to the organization of work at the healthcare centre, for example:

When she registers me, she immediately takes my documents to the doctor and I don't need to wait. (Participant 13)

## 5 DISCUSSION

This qualitative research study explored the qualities of a good family nurse from the perspective of older patients. The responses of our participants show, however, that the term “good family nurse” is less connected with her professional qualifications than with her expression of a positive attitude towards the patient, especially when the patient is older, a point which was often emphasized.

Elderly patients desire to be treated with respect; they want the nurse to support them, understand their problems and be nice and kind—ideas which were stressed by nearly all participants. Other qualitative research shows that, during a visit to the family doctor, older patients appreciate a doctor's socio‐emotional behaviours more than his/her medical competence (Marcinowicz, Pawlikowska, & Oleszczyk, 2014). Another study carried out among senior citizens reported that the participants highly valued a kind and open attitude in their doctors (Berkelmans, Berendsen, Verhaak, & Meer, 2010).

The categories and subcategories we determined in this original study are connected with the specificity of a family nurse's job and the organization and functioning of PHC. For example, the “availability” category was made up of two subcategories: home visits and the organization of family doctor's appointments. The participants identified some attributes of availability on the basis of observations of the work of a family nurse at the outpatient clinic (e.g. the organization of a family doctor's appointments), and others were the expression of their unmet expectations (e.g. more frequent visits of a family nurse).

In our research, one of the categories that defines a good family nurse is communicating with the patient. Participants talked about both good and bad experiences in communicating with the patient. They paid particular attention to the tone of the nurse's voice (“that she would not scream”) and her ability to listen (“that she would hear what I had to say”). They often emphasized that they are elderly people and expect the nurse to understand their situation.

Communication skills are a very important element of nursing care from the perspective of older patients, which is confirmed by the results of other studies. For example, in a study by Dahlke et al. (2018), older people and their families identified effective communication as a necessary component of providing care for older people, taking into account their needs.

In the “ethical behavior” category, patients often mentioned and stressed the nurse's respect for elderly people, openness to human suffering and a vocation to the nursing profession. Ethics is a basic component of nursing practice, and the development of ethical competence is an integral part of nursing education (Lechasseur, Caux, Dollé, & Legault, 2018). Family nurses need support in this competence, especially when they provide care for older patients.

In their literature review, Van der Elst et al. (2012) found that good nurses are defined by good technical and psychosocial skills; also, good nurses like their job and are understanding and caring. However, their review does not discuss the moral quality of nurses' behaviour in a broad ethical sense. In our research, ethical attitude was one of the categories that defined a good family nurse. Participants described this category mainly in terms of respect for the patient and openness to human suffering. As in the review by Van der Elst et al. (2012), the participants in our study said that a good family nurse should also like her job.

Although nursing has been evolving since the time of Florence Nightingale, our research shows that elderly patients perceive a good family nurse mostly in terms of her personality traits. Despite professional autonomy and academic development, Nightingale's statement that “You cannot be a good nurse without being a good woman”, to which other scholars also refer (Smith & Godfrey, 2002), still seems to be valid.

Understanding the characteristics of a good family nurse is important in strengthening the role of a nurse in PHC. The results of our research provide new information on personal attributes and ethical values that characterize a good family nurse. Qualitative research makes it possible to explore the term “good family nurse” on the basis of older patients' experiences and expectations as well as the vocabulary they use. Our research provides practical information on what qualities and skills a family nurse should have to meet patients' expectations of a “good family nurse.” Older patients are very sensitive to whether they are treated with respect and how the nurse approaches them. To improve the quality of care, family nurses, in addition to developing their professional competences, should improve their communication skills and present an ethical attitude. The results of this kind of research help improve nursing practice by professional training and the quality of health services, and they can facilitate the decisions made by older adults in choosing their family nurse. Moreover, they should be taken into consideration in the curricula of post‐graduate courses for family nurses.

### 5.1 Strengths and limitations

The strength of our study is that we selected the participants from among all the registered people receiving care of a family nurse, with various health problems, at six PHC providers. There are several limitations of the study that must be acknowledged. The data were collected at only one time‐point. In addition, we did not take into account caregivers' perspectives of what constitutes a good family nurse. The term “good family nurse” is complex and usually in content analysis the categories should be mutually exclusive, which was not always the case in our study.

## 6 CONCLUSIONS

The term “good family nurse” is a complex one and comprises many personal traits and attributes, values and behaviours. All of them are important from the perspective of older patients but none of them alone is sufficient. Ideally, the family nurse would combine all of those elements to meet the expectations of patients.

## CONFLICT OF INTEREST

No conflict of interest has been declared by the authors.

## AUTHORS' CONTRIBUTIONS

LM and ET: Planning and designing the study. ET: Interviewing the participants. LM and ET: Analysing the results. LM: Writing the manuscript. LM and ET: Editing and approving the final version.

## ETHICS APPROVAL AND CONSENT TO PARTICIPATE

The consent of the Bioethics Committee of the Medical University of Bialystok (No. R‐I‐002/290/2017) was obtained for the study.
